# Supplementary material for: Integrated Transcriptomic and Metabolomic Analysis Reveal the Underlying Mechanism of Anthocyanin Biosynthesis in Toona sinensis Leaves
Source: Int J Mol Sci. 2023 Oct 23;24(20):15459. doi: 10.3390/ijms242015459 (PMC10607221; doi:10.3390/ijms242015459)
Supplement: Supplementary file 1 [file ijms-24-15459-s001.zip › ijms-2638413-supplementary/Supplementary Files/Supplementary Figure S1.pdf]

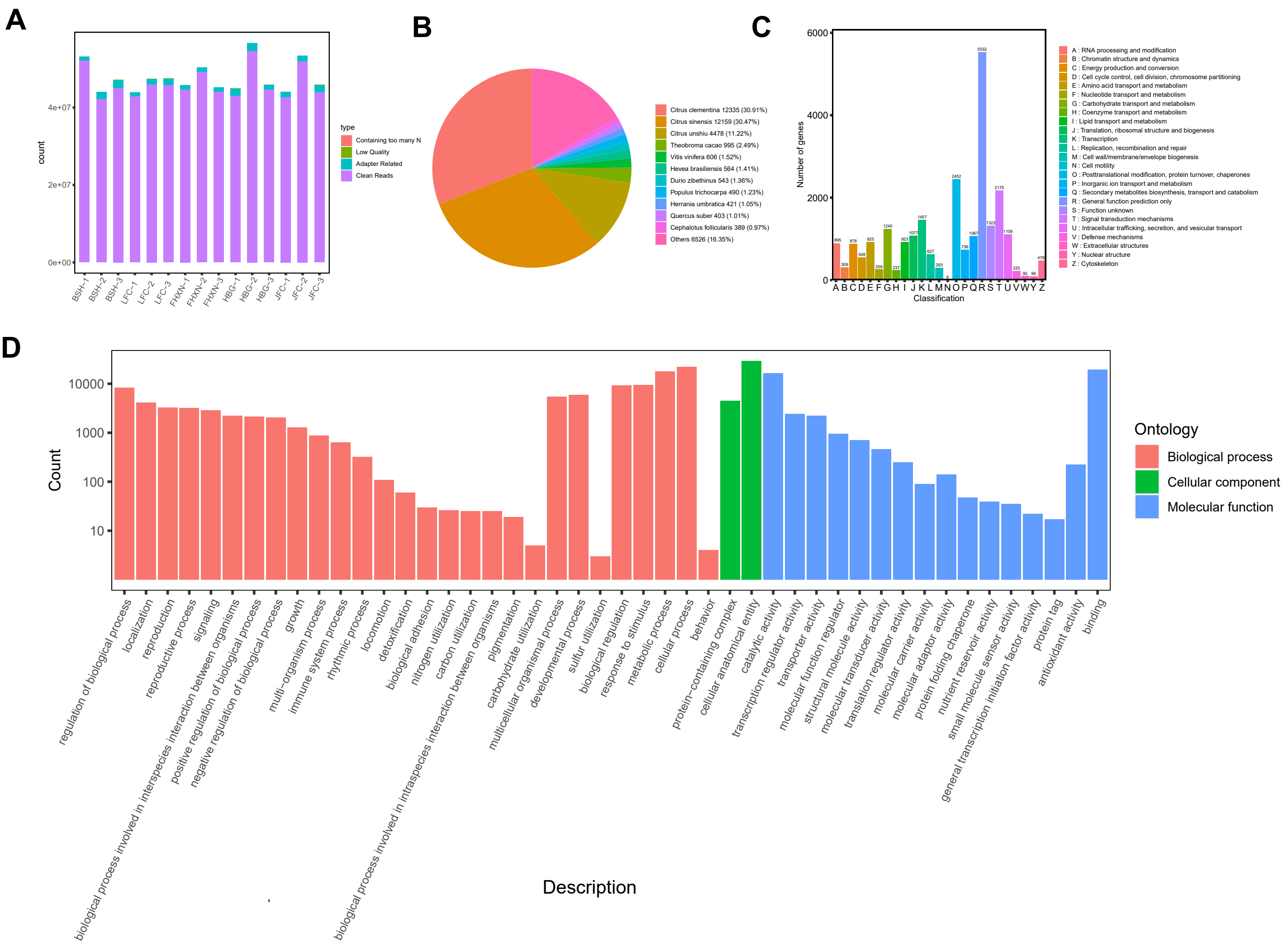

Supplementary Figure S1. Transcriptome data analysis and annotation.
(A) The count of reads.
(B) Compares the gene sequences of Toona with those of other varieties using NR database.
(C-D) The function term annotation of unigenes in KOG (C) and GO (D) databases.
