# Supplementary material for: Integrated Transcriptomic and Metabolomic Analysis Reveal the Underlying Mechanism of Anthocyanin Biosynthesis in Toona sinensis Leaves
Source: Int J Mol Sci. 2023 Oct 23;24(20):15459. doi: 10.3390/ijms242015459 (PMC10607221; doi:10.3390/ijms242015459)
Supplement: Supplementary file 1 [file ijms-24-15459-s001.zip › ijms-2638413-supplementary/Supplementary Files/Supplementary Figure S2.pdf]

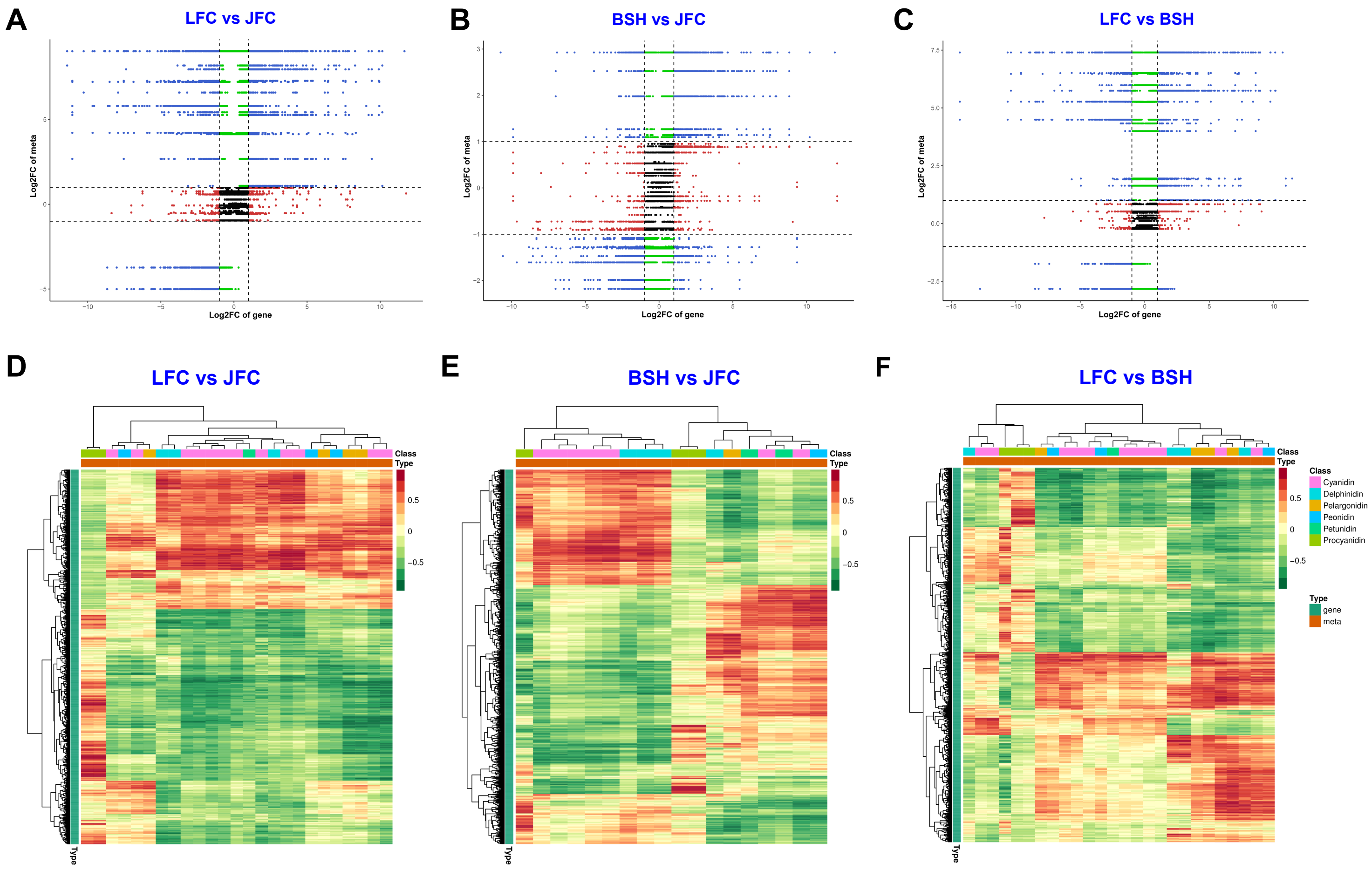

Supplementary Figure S2. Integrated analysis of transcriptome and metabolome among JFC (red), BSH (pink)，and LFC (green).

(A-C) The associations of the quadrant diagrams describing the transcriptomic and metabolomic variation for JFC/LFC, JFC/BSH, and BSH/LFC. (D-F) Heat maps of the correlation coefficient clusters (>0.8). (G) A KEGG pathway enrichment bubble plot for the DEGs found in JFC/LFC, JFC/BSH, and BSH/LFC groups.
