# Supplementary figures and images for: Integrated Transcriptomic and Metabolomic Analysis Reveal the Underlying Mechanism of Anthocyanin Biosynthesis in Toona sinensis Leaves
Source: Int J Mol Sci. 2023 Oct 23;24(20):15459. doi: 10.3390/ijms242015459 (PMC10607221; doi:10.3390/ijms242015459)

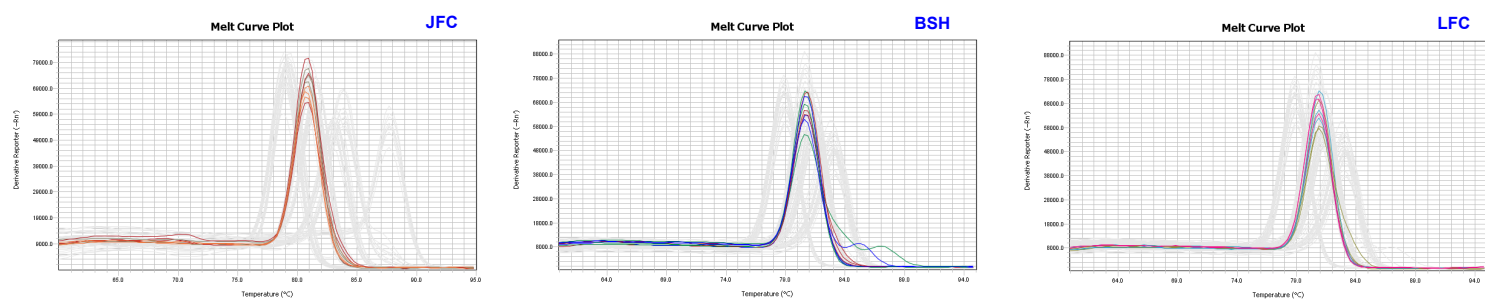

**Supplementary Figure S3. Melt curves from qRT-PCR of *ACT1N* gene in JFC, BSH, and LFC.**

Supplement: Supplementary file 1 [file ijms-24-15459-s001.zip › ijms-2638413-supplementary/Supplementary Files/Supplementary Figure S3.pdf]
